# Behind the Scenes of the Human Breast Cell Atlas Project

**DOI:** 10.1007/s10911-021-09482-7

**Published:** 2021-04-29

**Authors:** Renée van Amerongen

**Affiliations:** Developmental, Stem Cell and Cancer Biology, Swammerdam Institute for Life Sciences, University of Amsterdam, Amsterdam, the Netherlands

**Keywords:** Mammary gland, ScRNAseq, Human breast cell atlas, International consortium, Cell types, Population diversity

With the human genome project out of the way [1, 2] and the task of identifying all functional elements in our DNA bestowed upon the ENCODE consortium [3], scientists must have felt they were ready for the next big challenge: that of drafting a *magna carta* of the human body – and with single cell resolution at that. This is precisely what the Human Cell Atlas project aims to do. The overarching goal of the international consortium effort, backed by the Chan Zuckerberg Initiative (CZI) in the US, Horizon2020 in the EU and Wellcome in the UK, is to generate a collection of “cellular reference maps”, which will not only serve to catalogue the thousands of different cell types present in the human body, but which should also reveal cell state transitions, differentiation trajectories and lineage relationships.

I will confess that as someone not (yet) directly involved in the effort, it was not all that clear to me how things were progressing. In particular, I found it difficult to get a proper idea of the status of the Human Breast Cell Atlas, with scarce information seemingly limited to the CZI website. To increase visibility and interest in the project, which could be a game changer in our field, I therefore decided to use this special issue of the Journal of Mammary Gland Biology and Neoplasia as an excuse to contact some of the lead scientists involved in these pioneering stages. Luckily, they were more than willing to give me a behind the scenes glimpse of the past, present and future of the Human Breast Cell Atlas.

Following the launch of the Human Cell Atlas (HCA) at a kick-off meeting in London in October 2016, a white paper laid out its ambition and scope, emphasizing the need for standardization of experimental and computational methods [4]. The breast was notoriously absent from the initial list of biological systems presented by the founding consortium (as were other reproductive tissues), but this was to change a couple of months later, when Kai Kessenbrock (University of California, Irvine), Devon Lawson (University of California, Irvine), and Nicholas Navin (University of Texas MD Anderson Cancer Center) met at an HCA General Meeting, held at Stanford University in February 2017. Here, CZI introduced the project, its organization as a scientist-led consortium and its goals. “*At the poster session, we realized we were working in the same area, using single-cell tools to investigate cellular diversity in the breast, and planned to submit a multi-PI proposal to the upcoming pilot grant initiative funded by CZI*,” says Lawson. “*In our proposal, we planned to optimize protocols for breast dissociation and single cell sequencing, and proposed to generate a pilot atlas of the human breast.*”

With Navin as lead investigator and Kessenbrock and Lawson as co-principal investigators, they set out to produce a spatially resolved, multidimensional atlas using a diverse array of spatial and single-cell genomics technologies. “*Our labs have worked closely together to establish this framework and roadmap for how to build a comprehensive atlas of the human breast”*, explain the three scientists. The biggest achievements so far mainly concern the technical and administrative side of things: “*We have established an infrastructure as well as experimental best practices allowing us to build a comprehensive, spatially resolved atlas of the human breast using high-quality, normal breast tissue specimens. Also, we sought to meet highest ethical standards in the way the tissue donors will be consented, which required an iterative approach and close collaboration with our clinical collaborators and coordinators at UC Irvine and MD Anderson.”*

**Fig. 1:**
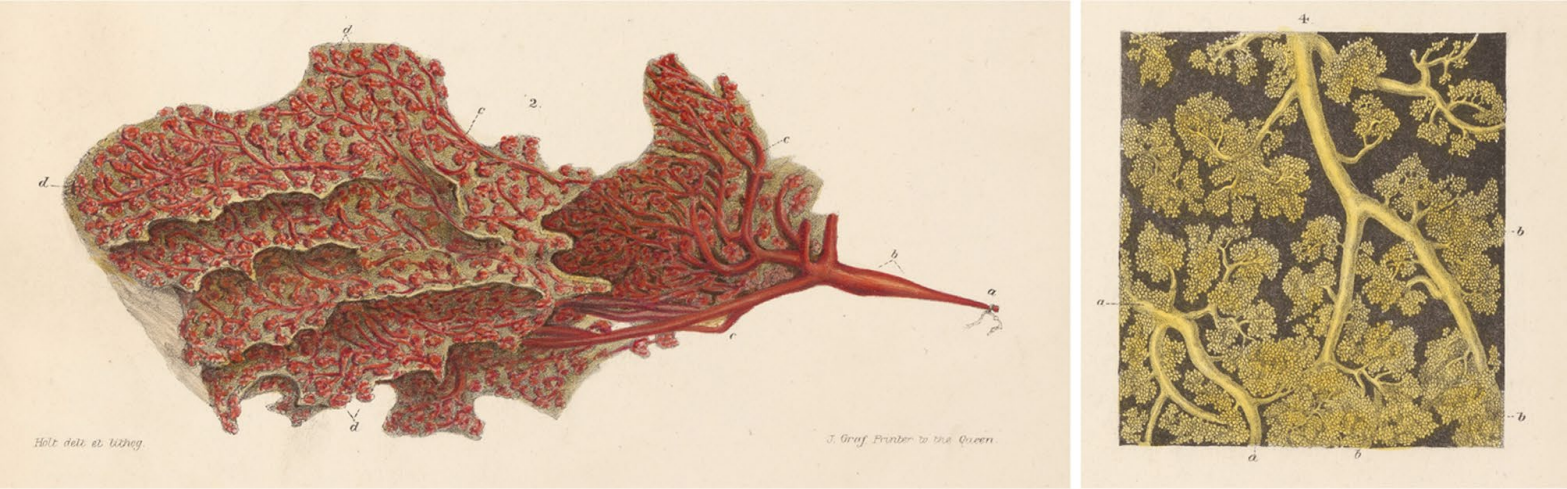
Arguably the most detailed atlas of the mammary gland available to date is that made by 19th century anatomist Sir Astley Paston Cooper (available via https://jdc.jefferson.edu/cooper/). The panels depicted here were taken from Plate VII, which shows “ducts, glandules and cells”. Shown are (left) a single lobule injected with red wax and (right) a close-up of a cluster of ducts and milk producing cells. The Human Breast Cell Atlas aims to map the composition of the human breast at single cell resolution, resulting in a catalogue of the cell state transitions, differentiation trajectories and lineage relationships of the different cell types in the human breast

But there is more – the investigators have also performed single-cell RNA, ATAC and single-nucleus RNA sequencing. On top of that, they have performed multiple types of spatial profiling, including protein (CODEX), and RNA (Spatial Transcriptomics and FISSEQ) in situ analyses. Although preliminary, these studies have already revealed some interesting findings. For instance, large numbers of adaptive immune cells can be found in and around both ducts and lobules. It will be exciting to learn more about how and when these cells localize there and what they are doing exactly. Another surprise was the epithelial diversity encountered. “*We observe three epithelial cell types, one basal and two luminal, as well as substates with each cell type. These cell types and states don’t fully reflect previous notions of mammary epithelial hierarchy, and we have yet to find a substate that resembles a stereotypical adult stem cell.*” The high-resolution characterization of lesser studied stromal populations, such as adipocytes, fibroblasts, pericytes and other cells types, with single-cell gene signatures for each, will also offer exciting new leads (Fig. 1).

In 2019, the Human Breast Cell Atlas Seed Network received additional funding from CZI, with Ken Chen and Bora Lim (also from the University of Texas MD Anderson Cancer Center) and Je Lee (Cold Spring Harbor Laboratory) joining as co-principal investigators. This should bring the goal of collecting data from more than 100 individuals representing diverse demographics and covering the key stages of the adult biological variation of the tissue (parity, menstrual cycle stages, menopause, etc.) within reach. The network of collaborators and advisors is also rapidly expanding, involving scientists from across the United States, including Zev Gartner (University of California San Francisco), Harikrishna Nakshatri (Indiana University), Yunlong Liu (Indiana University), Anna Maria Storniolo (Indiana University), Jared Haun (University of California Irvine), Long Cai (Cal Tech), Oliver Braubach (Akoya Biosciences), Thea Tlsty (University of California San Francisco) and Joan Brugge (Harvard).

Like any atlas, the HCA ultimately needs to cover the full globe. This is where the research team of Harikrishna Nakshatri comes in. “*Our collection contains tissues of women of different genetic ancestry, which allowed us to include tissues from diverse populations for the study.*” The tissue bank at Indiana University is indeed unique, as it contains samples from more than 5000 healthy women who have donated breast tissue specifically for research purposes. A couple of years ago, these tissues were used to demonstrate that commonly used “normal” controls from reduction mammoplasty or tumor-adjacent tissues were, in fact, histologically abnormal [5]. “*These observations prompted us to use normal breast tissues from our bank to generate a healthy breast atlas*”, says Nakshatri. Thanks to the existing infrastructure and a rapid tissue collection protocol, a total of 11 fresh or rapidly cryopreserved biopsies have already been subjected to scRNAseq, revealing 23 epithelial cell clusters [6]. Using tissues from core needle biopsies comes, quite literally, at a cost as the cellular content invariably differs between samples and the success rate of obtaining high quality data was less than 50 %. The COVID-19 pandemic has also thrown a spanner in the works as fresh tissue collection has been put on hold, leaving only cryopreserved samples to be used for, according to Nakshatri’s expectations, at least another year. “*Since age, parity, BMI, environment and genetic ancestry can influence biology of the breast, identifying core breast epithelial cell types that are not influenced by these confounding factors will be a challenge*”, Nakshatri is also quick to add.

What some see as confounding factors, others see as inherent dynamic properties of the tissue. Walid Khaled (Cambridge University, UK) became involved in the HCA in 2018, after publishing a scRNAseq study charting the differentiation dynamics of the mouse mammary gland [7] and responding to a grant call from the Medical Research Council together with John Marioni and Carlos Caldas (both at the Cancer Research UK Cambridge Institute). “*Our project aims to chart the cellular changes induced by aging, parity, lactation and germline mutations*”, explains Khaled. To date, his lab has completed the lactation study [8]. When asked about the challenges he encountered so far, the first thing that comes to mind is setting up a reproducible workflow for processing all the samples in a similar fashion. *“This took us nearly one year, but it was totally worth it as we now have a solid protocol and also a clear structure for what to do with each piece of data.”* That’s not all, however. *“I would also add that the metadata is equally important to (if not more important than) the sequencing data itself.”* This sentiment is echoed from across the pond. *“Metadata collection (parity, menstrual stage status, age, BMI, etc.) has been challenging, but it is essential to fully analyze and interpret data*”, agree Kessenbrock, Lawson and Navin, who also recognize the struggle of determining best practices for tissue processing, or, more specifically, of “*finding the sweet spot for tissue dissociation so that all cell types are captured while minimizing unwanted technical variation and stress responses”.* Both the US and the UK teams have also had to overcome in silico issues. *“Computational analysis has required an iterative process of filtering out technical artefacts (doublets, dead cells, ambient RNA, etc.)”*, explain Kessenbrock, Lawson and Navin. Next, Khaled and Nakshatri suspect, comes the difficulty of integrating the datasets from multiple labs. This is the only way to ensure that the current results are reproducible and capable of identifying bona-fide changes that are not driven by batch effects or biological variability between individuals. In the end, a meta-analysis will be needed to identify widely applicable biomarker sets, thinks Nakshatri.

But there are other challenges as well. Organization of a large multi-group project takes extensive planning, time, and commitment. The US team meets twice per week to discuss data analysis and plan next steps. They add: *“Another challenge is data transfer between sites, and we had to make significant effort to organize transfer of sequencing and spatial data across campuses. Building an ethical method for consenting patients has been a major an ongoing discussion by HCA and CZI as well as within our own group. A major goal of the consortium is to make all data publicly available. Since it has become increasingly possible to identify individuals and family members from sequencing data, protections of privacy and fully informed consent are paramount.”*

**Fig. 2:**
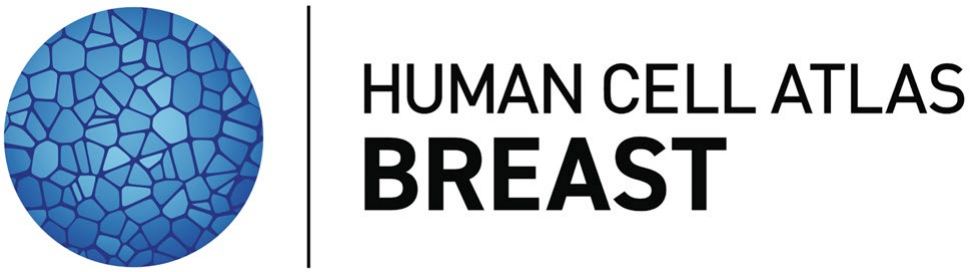
Official logo of the Human Breast Cell Atlas. Scientists interested in joining the network and attending any of the biological network seminar series can register their interest on the HCA website at https://www.humancellatlas.org/areas-of-impact/

Now that the Human Breast Cell Atlas has been placed on the map, so to speak, the next logical question is if and how the rest of the international mammary gland and breast cancer community can get involved (Fig. 2). Here, there is plenty of work to be done. Take, for instance, the issue of nomenclature for different cell types and cell states that are going to be uncovered, which, Kessenbrock, Lawson and Navin agree, will require a community effort. With multiple Human Breast Cell Atlas projects going on across the globe, those interested to become actively involved in any of the working groups are encouraged to check out https://www.humancellatlas.org/areas-of-impact/, where information on the various HCA initiatives, such as the biological network seminar series, can be found. Colleagues who are organizing conferences and meetings should also take note: *“A great way to get real tangible input from the community could be to host breakout or brainstorming session at upcoming breast meetings, such as the Gordon Conference for Mammary Gland Biology or the AACR breast cancer meetings,”* suggest Kessenbrock, Lawson and Navin.

With so many technological possibilities available in the lab and others on the horizon, I personally think the input from mammary gland biologists, anatomists, epidemiologists and others with relevant biological expertise remains invaluable and perhaps even critical for success – especially when it comes to making the choices that ultimately underlie sample selection. As such, a stand-alone human breast cancer atlas symposium or workshop would also make sense. Nakshatri, in addition, sees golden opportunities for bioinformatics specialists, as new tools for the analysis of single-cell data are constantly being published. “*Every publication claims that their tool outperforms other tools, but an unbiased analysis by independent investigators would benefit all.”*

Ultimately, the Human Breast Cell Atlas aspires to provide a resource for the global scientific community. *“We aim to provide reliable, accessible and easy to interpret datasets for the community to utilize as the starting point for their research questions”*, says Khaled. The HCA consortium actively promotes open access of data and protocols, including rapid and data dissemination through pre-prints. The latter, suggests Nakshatri, also offers researchers worldwide the opportunity to give input on projects and even to suggest alternative interpretations of the data. An online portal from which all datasets and the aforementioned metadata will be downloadable is currently under construction. It will contain a web interface to facilitate analyses. Constructing this portal is a substantial effort in and by itself, which is being organized by a group of data wranglers.

Excitingly, the Human Breast Cell Atlas network is also in the process of building a smaller, more focused portal (to be launched at https://hbcaportal.org) that will hopefully be of great value to the wider mammary gland and breast community. Kessenbrock, Lawson and Navin elaborate: “*The portal will contain information about the goals and scope of the project, as well as how labs can contribute. We will post links for dataset downloads, and create an interactive web software interface so that users can perform some basic analyses without having to download datasets or have experience with coding. We are modeling it after other online portals, such as KM plotter* (https://kmplot.com/analysis/) *and The Mouse Cell Atlas* (http://bis.zju.edu.cn/MCA/)”. As someone on the user end, I hope that such a portal will include intuitive access to multiple bioinformatics tools, while also allowing high-resolution, customizable export of graphs and analyses to promote their inclusion in publications by many.

But it is not all about accessibility. “*A complete atlas requires the generation of spatial maps across multiple scales. One of the main challenges is to bridge and connect between macro-, meso- and micro-scale information. It is relatively easy to map cells using histology on the microscale, but it is very challenging to build spatial models of the breast that are accurate at the meso- (e.g. connected lobular units, larger connective and lactiferous ductal systems) and macroscale (i.e. different anatomical regions of the breast such as quadrants, nipple area, etc.*)”, explains Kessenbrock. Once this is achieved, however, the Human Breast Cell Atlas should serve as a reference for the whole research community to understand how cells, cell niches, cell communities and cell-cell communication become deregulated to result in breast cancer and disease. Ultimately, this should enable breast cancer researchers and physicians to detect much earlier when the tissue deviates from the normal spectrum of cellular patterns, and as such improve methods for cancer early detection and, given enhanced knowledge about the cells of origin for different subtypes, for better treatment. Yet even without these important clinical implications it is clear that exciting times lie ahead as we are about to uncover fundamental properties of cell growth, differentiation and developmental dynamics of the mammary gland. After almost 200 years, Sir Astley Paston Cooper’s “On the anatomy of the breast” [9] may finally get a worthy successor.
